# Audit of pharmacological management of borderline personality disorder as per NICE clinical guidelines CG78

**DOI:** 10.1192/bjo.2021.841

**Published:** 2021-06-18

**Authors:** Bethany Dudley, Shakina Bellam, Andrew Lawrie

**Affiliations:** 1 Monkwearmouth Hospital, Cumbria Northumberlnd Tyne, Wear NHS Foundation Trust; 2 Monkwearmouth Hospital, Sunderland, Cumbria, Northumberland Tyne Wear NHS Foundation Trust

## Abstract

**Aims:**

To audit the current practice of pharmacological management of Borderline Personality Disorder with NICE Clinical guideline [CG78]: Borderline personality disorder:

Objectives:

23 patient records were analysed in the last 18months with a diagnosis of EUPD to compare current practice against NICE clinical guidance. (2009)

Standards:

When prescribing

1. Use a single drug.
2. Use the minimum effective dose.
3. Agree with the person the target symptoms, monitoring arrangements and anticipated duration of treatment. Antipsychotic drugs should not be used for medium, long term treatment.
  Indication:
4. Drug treatment should not be used specifically for borderline personality disorder or for the individual symptoms or behaviour associated. (Repeated self-harm, marked emotional instability, risk taking behaviour and transient psychotic symptoms).
5. Short-term use of sedative medication may be considered cautiously as part of the overall treatment plan in a crisis. The duration of treatment should be no longer than 1 week.
6. When considering drug treatment, provide the person with written material about the drug. This should include evidence for the drug's effectiveness in the treatment of borderline personality disorder and for any comorbid condition, and potential harm.
  Review:
7. Review the effectiveness and tolerability of previous and current treatments.
8. Discontinue ineffective treatments.

**Background:**

Borderline Personality Disorder is common in psychiatric settings with a reported prevalence of 20%.

As per NICE Guidance (CG 78), no medications have been found effective for the longer term treatment of personality difficulties.

This audit was carried out to review if patients were offered psychiatric reviews to discuss the medications they are using, the effectiveness of these, and any potential side effects.

**Result:**

Good practice compliance of 90-100% was noted where >90% compliance was seen in areas where the effectiveness and tolerability of current and previous medication was reviewed by the clinicians under Structured Clinical Management. Also was noted that antipsychotics were not used for medium to long term in patients with Borderline Personality Disorder in the cohort.

The following areas were non-compliant with the NICE recommendations where a compliance <79% has been achieved.

When prescribing, use a single drug (avoid polypharmacy), agree target symptoms, monitoring and duration, provide written information, discuss evidence for effectiveness in treatment of borderline personality disorder.

Partial compliance was achieved (80-89%) with use of sedatives for less than 1 week and discontinuation of ineffective treatment.

**Conclusion:**

Distribute key cards to clinicians.

Provide written information to patients.

Re-audit in 6 months.

